# Selective screening for familial hypercholesterolemia in Austrian children - first year results

**DOI:** 10.1186/s12887-019-1586-4

**Published:** 2019-06-25

**Authors:** Alexandra Kreissl, Nina Walleczek, Pinky Rose Espina, Ulrike Hallwirth, Susanne Greber-Platzer

**Affiliations:** 1 Division of Pediatric Pulmonology, Allergology and Endocrinology, Department of Pediatrics and Adolescent Medicine, Medical University of Vienna, Waehringer Guertel 18-20, 1090 Vienna, Austria; 2 Municipal Authority of the City Vienna, Municipal Department 15, Health Service of the City of Vienna, Vienna, Austria

**Keywords:** Familial hypercholesterolemia, Selective screening, Cholesterol, LDL cholesterol, Children

## Abstract

**Background:**

Familial hypercholesterolemia (FH), the most frequent monogenetic hereditary disorder, is underdiagnosed and undertreated. Early identification of FH is essential because of the increased risk for premature cardiovascular diseases and childhood might be the optimal period for cholesterol screening. Aim of this selective screening was to detect familial hypercholesterolemia, the most frequent monogenetic hereditary disorder in children to guarantee early detection and treatment. The Austrian strategy for primary schools, to perform a pre-school examination by school physicians, allows to reach all children aged 5–7 years.

**Methods:**

The screening was conducted within the school enrolment examinations in all 215 public primary schools in Vienna between January to May 2017. Positive cholesterol screening was defined by non-HDL-C > 160 mg/dL and/or LDL-C > 130 mg/dL.

**Results:**

In total, 18,152 children had their school enrolment examination. From 133 tested pre-school children, nine individuals were positive-screened with a mean LDL-C of 161 ± 26 mg/dL, non-HDL-C of 181 ± 24 mg/dL and total cholesterol (TC) of 239 ± 23 mg/dL. From 85 siblings, four individuals were positively screened with a mean LDL-C of 150 ± 7 mg/dL, non-HDL-C of 184 ± 8 mg/dL and TC of 231 ± 10 mg/dL. Patients did not have any xanthomas, xanthelasms, arcus lipoides, or any cardiovascular comorbidities.

**Conclusions:**

Screening at early childhood by school physicians seems to be a successful strategy and possible. With this Austrian selective screening method, FH Kids Austria, we could find nine patients with positive raised level LDL-cholesterol and/or non-HDL cholesterol out of 133 blood tests. Prevention of cardiovascular diseases is essential and it is our duty to increase the awareness of this disease. Limitations of the FH Kids project were reduced participation of school physicians and refusal of the parents.

## Background

Familial hypercholesterolemia (FH) is the most frequent monogenetic hereditary disorder and a common cause of premature cardiovascular diseases (PCVD) [1]. The relationship between morbidity and mortality to early treatment seems to be essential [1, 2]. Therefore, early detection and strict therapy are necessary to prevent PCVD. Heterozygous familial hypercholesterolemia (heFH) is an autosomal dominant disease. FH is characterized by markedly elevated plasma concentrations of low density lipoprotein cholesterol (LDL-C), which are already present at birth [3]. FH is caused by genetic mutations of the LDL-receptor (LDL-R), Apolipoprotein B (ApoB), proprotein convertase subtilisin/kexin type 9 (PCSK9) or low-density lipoprotein receptor adaptor protein 1 (LDLR-AP1) [4]. The prevalence of heterozygous familial hypercholesterolemia is between 1 per 250 in the European population [5–7]. The prevalence of homozygous familial hypercholesterolemia (hoFH) is between 1 per 160,000 to 300,000 [5–8].

Familial hypercholesterolemia is underdiagnosed and undertreated according to the following facts: i) the disease and its high frequency is still largely unknown among the general population, ii) missing symptoms in patients, especially in children and adolescents, and iii) screening strategies are globally inconsistent or even not available. Three types of screening can be distinguished: the selective screening, cascade screening and the universal screening. Selective screening for FH is based on the measurement of cholesterol levels in children, with a positive family history for hypercholesterolemia or premature cardiovascular disease in first- or second-degree relatives [9–11]. Cascade screening can describe a direct cascade screening or a reverse cascade screening. Direct cascade screening is based on the known genetic defect for a disease of an individual, indicating genetic testing in all first-degree relatives. This type of screening is the most cost-effective strategy for FH [12, 13]. Reverse cascade screening is based on the fact that a child is the index patient, followed by testing in the parents and other relatives [14]. In universal screening a nationwide measurement for example of cholesterol is performed in a certain age group of childhood [15, 16].

The aim of the study, FH Kids Austria, was to detect FH in young aged children and to guarantee early detection and treatment. The Austrian strategy for primary schools, to perform a pre-school examination by school physicians, allows to reach all children aged 5 to 7 years and their parents. Therefore, a selective screening strategy for familial hypercholesterolemia was considered as most suitable and as considered feasible by school physicians.

## Methods

### Study design

This prevention-study was implemented to identify children with familial hypercholesterolemia with a selective screening in primary school children aged between 5 to 7 years. The screening was conducted within the school enrolment examinations in all 215 public primary schools in Vienna between January to May 2017. The screening was based on three questionnaires, the school physician questionnaire about the child and a questionnaire for each parent available in German, English, Turkish and Bosnian-Croatian-Serbian. Each questionnaire included three short questions and thus many negative families were able to be eliminated for screening.

Questionnaire:

1) Do you (biological mother or father) or close relatives (siblings, grandparents, aunts, uncles) have elevated blood lipids (= total cholesterol, triglycerides, LDL-cholesterol) or does someone take cholesterol-lowering medication (statins)?

O Yes O No if yes: please specify on you / your relatives:

2) Do you (biological mother or father) have fatty skin lumps (=xanthomas) in particular on the areas of the Achilles tendon/ hands/knees or eyes (=xanthelasma)?

O Yes O No.

3) Did you (biological mother or father) or close relatives (siblings, grandparents, aunts, uncles) suffered a heart attack or stroke before the age of 55?

Parents were contacted if one of the questions were positively answered, not-answered or unknown answers (e.g. foster or adopted child) were provided.

### Cholesterol level determination

Cholesterol screening test was performed with the Alere AfinionTM AS 100 Analyzer (Alere GmbH, Linz, Austria) at the Department of Pediatrics and Adolescent Medicine at the Medical University of Vienna from January to September 2017. The cholesterol screening test was taken from a capillary blood sampling from a puncture on the finger. The measured blood parameters included total cholesterol (TC), high-density lipoprotein cholesterol (HDL-C), and triglycerides (TG). Low-density lipoprotein cholesterol (LDL-C), non-HDL cholesterol (non-HDL-C), and cholesterol/high-density lipoprotein cholesterol ratio (C/HDL-C ratio) were calculated by the device. The cut-off level for a positive cholesterol blood screening was defined either as strong positive by non-HDL-C > 190 mg/dL and/or LDL-C > 160 mg/dL or as borderline by non-HDL-C > 160 mg/dL and/or LDL-C > 130 mg/dL. The pre-school children as well as their siblings were tested. Positive screened children as well as individuals were invited to follow the standardized familial hypercholesterolemia program at the outpatient clinic of obesity, lipometabolic disorder and nutritional medicine at the Department of Pediatrics and Adolescent Medicine. The study was approved by the local Ethics-Committee at the Medical University Vienna (MUV, EC Nr: 2019/2015). All parents were informed and provided their written informed consent.

### Statistical methods

Metric variables are shown as median, range and mean with SD and dichotomous variables were described by absolute and relative frequencies. All statistical analyses were performed using the software Statistical Package for Social Science for Windows version 24 (SPSS Inc., Chicago, USA).

## Results

### Study population

**Fig. 1:**
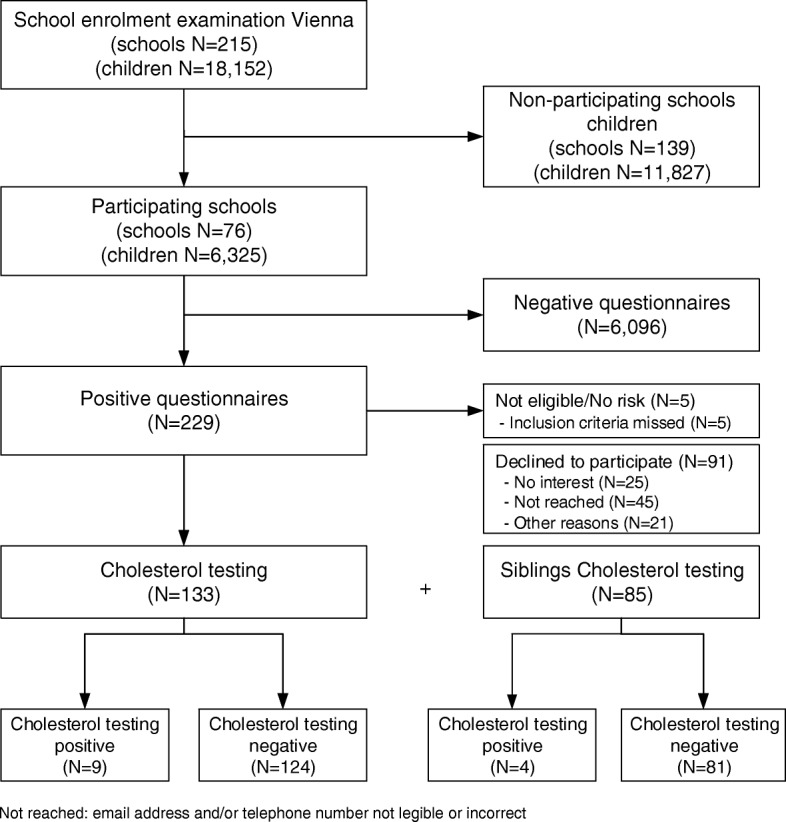
Flow Chart of a Selective FH-Screening in Austrian Children

A total of 18.152 children had their school enrolment examination, 6.325 answered the questionnaires and 229 provided positive questionnaires, defined as questions which were positively answered, not-answered or unknown answers. Finally, 133 pre-school children came to the clinic for the lipid screening test (Fig. 1). Vienna consists of 23 districts and the majority of the tested pre-school children came from the second district (Leopoldstadt) (15.4%), third district (Landstraße) (15.4%) and the 12th district (Meidling) (10%). Biological mothers were from 20 different countries of birth and biological fathers from 23 different countries of birth. The majority came from Austria, with the same percentage of 41.4% in both parents, followed by Turkey (mothers: 9.8%, fathers: 11.3%). The mothers age was 36 years (=median, range: 22–50 years) and fathers were 39 years (=median, range: 23–53 years) old. An amount of 22% of mothers and 35% of fathers indicated in the questionnaire to suffer from hypercholesterolemia. Xanthomas and xanthelasma were present in 2.3% mothers and in 3.8% fathers respectively. More fathers (9.8%) than mothers (3.8%) suffered from coronary heart disease (CHD). More first- and second-grade relatives of mothers (60.2%) than of fathers (40.6%) suffered from hypercholesterolemia. A premature coronary heart disease has been determined in 37.6% of maternal close relatives and in 17.3% of paternal close relatives. The pre-school children had up to three siblings. The flow diagram visualizes the implementation of the screening (Fig. 1).

### Patient characteristics

The demographic data are presented in Table 1. In 11% of the pre-school children, the following diseases have been noticed: obesity (*n* = 5), allergy (*n* = 2), neurodermatitis (*n* = 1), obstipation (*n* = 2), iron deficiency (*n* = 1), kidney disease (n = 1), epilepsia (*n* = 1) and myositis (*n* = 1). Clinical symptoms of FH were not determined in these children.

**Table 1:** Demographic characteristics

	Pre-school Children (*N* = 133)	Index patients Positive (*N* = 9)	Siblings All (*N* = 85)	Siblings Positive (*N* = 4)
	Median (Range)	Median (Range)	Median (Range)	Median (Range)
Male	73 (55%)	6 (67%)	31 (37%)	1 (25%)
Age (years)	6 (5–7)	6 (5–6)	8 (0.5–28)	2 (0.5–12)
Body height (cm)	118 (100–140)	119 (110–127)	134 (68–190)	88.5 (68–160)
Body weight (kg)	22 (14–49)	22 (20–27)	31 (7–88)	11 (7–42)
BMI (kg/m^2^)	15.8 (10.7–30.7)	16.0 (15.3–16.7)	16.5 (8.9–34.7)	14.5 (13.5–16.4)

Data are presented as median and range, or as number of subjects (%)

*BMI* Body Mass Index

### Cholesterol screening test

Out of the 133 pre-school children, nine individuals were positively screened with a mean LDL-cholesterol level of 161 ± 26 mg/dL, non-HDL cholesterol of 181 ± 24 mg/dL and mean total cholesterol level of 239 ± 23 mg/dL. This means that 3 patients were strong positive with a mean LDL-C of 193 ± 17 mg/dL, non-HDL-C of 208 ± 17 mg/dL and total cholesterol (TC) of 236 ± 26 mg/dL and 6 patients borderline with a mean LDL-C of 146 ± 10 mg/dL, non-HDL-C of 168 ± 13 mg/dL and TC of 227 ± 8 mg/dL. In 85 siblings the lipid screening test was performed and four individuals were positively screened with a mean LDL-C of 150 ± 7 mg/dL, non-HDL-C of 184 ± 8 mg/dL and a mean TC of 231 ± 10 mg/dL (Table 2). In all groups, no gender differences have been observed according to the screening lipid test.

**Table 2:** Cholesterol screening test

	Pre-school Children (*N* = 133)	Index patients Positive (*N* = 9)	Sibling All (*N* = 85)	Siblings Positive (*N* = 4)
	Median (Range)	Median (Range)	Median (Range)	Median (Range)
TC (mg/dL)	155 (99–281)	233 (218–281)	152 (99–238)	234 (217–238)
LDL-C (mg/dL)	76 (30–210)	152 (129–210)	70 (22–157)	150 (141–157)
HDL-C (mg/dL)	57 (22–100)	54 (42–80)	51 (25–101)	48 (27–66)
TG (mg/dL)	87 (44–493)	82 (47–176)	118 (44–491)	156 (118–247)
non-HDL-C (mg/dL)	97 (52–225)	181 (150–225)	99 (51–190)	186 (172–190)
C/HDL ratio	2.7 (1.6–6)	4 (3–6)	3 (1.6–8)	5 (4–8)

Data are presented as median and range. Cholesterol screening test was performed by capillary blood sampling

*TC* Total Cholesterol, *LDL-C* Low Density Lipoprotein Cholesterol, *HDL-C* High Density Lipoprotein Cholesterol, *TG* Triglycerides, *non-HDL-C* non-HDL Cholesterol, *C/HDL ratio* Cholesterol/High Density Lipoprotein Ratio

After four months the cholesterol screening test from the capillary blood sampling was confirmed with venous blood sampling. No significant differences were found between the two methods. Table 2 summarizes the blood results from positive screened index patients. All positive screened index patients and positive screened siblings had a positive family history according to hypercholesterolemia in one parent and grandparent, but neither showed any other disease related with dyslipidemia nor presented xanthomas or xanthelasma, arcus lipoides, or atherosclerotic comorbidities. In four index patients and one positive screened sibling results for familial hypercholesterolemia showed a disease causing genetic mutation either on LDL-R or on ApoB.

### Sensitivity and specificity

Based on familial hypercholesterolemia prevalence of 1:250 [5–7] and with the assumption that all children with positive questionnaires would have been included in the cholesterol testing (*n* = 229), the sensitivity and specificity of the selective FH-screening has been evaluated. For a positive cholesterol screening test the sensitivity is 60% and specificity is 97% (Table 3).

**Table 3:** Sensitivity and Specificity of a selective FH-Screening

Prevalence	Sensitivity (%)	Specificity (%)	PPV (%)	NPV (%)
1:250	60	97	6.55	99.84

Data are presented as percentage. *PPV* Positive Predictive Value, *NPV* Negative Predictive Value

## Discussion

In the current study, we have investigated the first screening for familial hypercholesterolemia in children from Austria. Various screening methods are recommended for different paediatric age groups. The American Academy of Pediatrics recommended a universal screening in children aged between 9 to 11 years, and re-screening at the age between 17 to 21 years [17]. Universal screenings were performed in five year old children in Slovenia [18] and in one to two year old children in the UK [19] at routine child immunisation visits. In Slovakia universal screening was conducted in children aged between 11 to 17 years during cholesterol screening visits [20]. In Austria routine medical checks in children are often not perceived by the parents and therefore, universal screening cannot reach the population. In consequence, we tried to establish a screening method for children at a time point, which assured to reach this young study population all over Vienna. In Vienna’s public primary schools, annually repeated school enrolment examinations for pre-school children are performed by school physicians. Prior to the start of the study all school physicians were trained for this screening. We gave up universal screening because of limited time resources of the school physicians and legally problematic presence of scientific staff at the school enrolment. The prevalence of heFH is estimated approximately one per 250 in the general population [5–7]. From 133 tested pre-school children, nine individuals were positive-screened. From 85 siblings, four individuals were identified to be positive. Therefore, the school enrolment examination might be an optimal time to reach all young aged children and to identify FH children in Austria. It would be an additional advantage that the knowledge of FH among all parents of pre-school children and the school physicians may improve, who are often primary care physicians, which helps to increase the FH awareness. Early identification of FH patients is essential and childhood is described as the optimal period for cholesterol screening [21]. During adolescence, the relatively reduced LDL-cholesterol may be problematic for a screening strategy [22, 23]. Hence, a screening in childhood might be an appropriate and most effective screening to detect those patients. First line treatment consists of fat reduced diet and physical activity, but if necessary medication can be started from the age of 6–10 years [1, 2].

In fact, the majority of patients suffering from FH are undiagnosed until their first often dramatic cardiovascular event [6]. How could that be changed?

The implementation of lipid screening in children has the strength to recognize FH and to prevent early atherosclerotic cardiovascular disease (ASCVD) [24], but also to identify other lipid disorders like obesity or combined dyslipidemia [25]. Patients suffering from familial hypercholesterolemia have a 100-times increased PCVD risk [26]. This was confirmed in the study by premature CHDs in first- and second-grade relatives. Early treatment is cost-effective in terms of cost per year of life saved [1] and should be implemented in high risk children to lower LDL-C. For the cholesterol screening test the Alere AfinionTM AS 100 Analyzer (Alere GmbH, Linz, Austria) has been used. For this kind of measurement only one drop of blood is needed, received from a finger stick. The benefits are that the measurement is fast and can be taken non-fasting by measuring directly TC, HDL-C, TG and additional calculation of LDL-C, non-HDL-C and C/HDL ratio. Moreover, for confirmation of the FH diagnosis in the patients’ clinical criteria including family history, lipid status and genetic testing were used. Thereby, nine index patients could be detected in early childhood, which guarantees an excellent quality of life by strict lipid lowering therapy. In four index patients and also one positive screened sibling the diagnosis was genetically confirmed by either a pathologic LDL-R mutation or on ApoB mutation.

With the assumption that all children with positive questionnaires would have been included in the cholesterol testing based on the positive predictive value (9/133) of 6.77%, a total number of 15 positive tested children could be detected, with a sensitivity of 60% and a specificity of 97%. The sensitivity and specificity of the selective screening has been determined based on a prevalence of 1:250 [5–7]. However, the Austrian prevalence data of heFH is not available. Hence, previous published data on prevalence of 1:500 [27], would result into an increased sensitivity of this FH screening method. Therefore, this shows the potential which might be achieved with selective screening in early childhood. However, this is the first time of a screening method for FH in Austria and could show the high potential of finding affected children. Almost certainly universal screening during the school enrolment would increase the detection rate.

A limitation of the study was that we received questionnaires only from 35% of all Viennese children, who had their school enrolment examinations. Reasons not to be included into the screening were that school doctors had no sufficient time resources, little experience with studies, parents refused study participation and despite multilingual study documents language difficulties seemed to be the main factors, which may affect the outcomes of sensitivity and specificity for this heFH screening tool. From the positive questionnaires, almost 20% of parents could not be contacted as email address and/or telephone numbers were not legible or incorrect. Those aspects have to be improved for further screening periods as well as to increase the awareness of parents and school physicians for selective FH screening.

## Conclusions

Early detection and prevention of cardiovascular diseases are essential for the commonly frequent genetic disease, familial hypercholesterolemia. Screening at early childhood by school physicians seems to be a successful strategy and possible. With this Austrian selective screening method, FH Kids Austria, we could find nine patients with strong positive or borderline LDL-cholesterol and/or non-HDL cholesterol out of 133 blood tests. Prevention of cardiovascular diseases is essential and it is our duty to increase the awareness of this disease. Limitations of the FH Kids project were reduced participation of school physicians and refusal of the parents. Therefore, the school enrolment examination might be a suitable strategy to identify FH children in Austria.

## Data Availability

The datasets used and/or analyzed during the current study are available from the corresponding author on reasonable request.

## Abbreviations

ApoB: Apolipoprotein B
ASCVD: Atherosclerotic Cardiovascular Disease
C/HDL-C ratio: Cholesterol/High-Density Lipoprotein Cholesterol ratio
CHD: Coronary Heart Disease
FH: Familial Hypercholesterolemia
HDL-C: High-Density Lipoprotein Cholesterol
heFH: Heterozygous Familial Hypercholesterolemia
hoFH: Homozygous Familial Hypercholesterolemia
LDLAPR1: Low-Density Lipoprotein receptor Adaptor PRotein 1
LDL-C: Low Density Lipoprotein Cholesterol
LDL-R: LDL-Receptor
non-HDL-C: Non- High-Density Lipoprotein Cholesterol
PCSK9: Proprotein Convertase Subtilisin/Kexin type 9
PCVD: Premature Cardiovascular Diseases
SPSS: Statistical Package for Social Science
TC: Total Cholesterol
TG: Triglycerides
